# Electrical impedance imaging system using FPGAs for flexibility and interoperability

**DOI:** 10.1186/1475-925X-13-126

**Published:** 2014-08-30

**Authors:** Harsh Sohal, Hun Wi, Alistair Lee McEwan, Eung Je Woo, Tong In Oh

**Affiliations:** Department of Biomedical Engineering and Impedance Imaging Research Center, Kyung Hee University, 446-701 Yongin, Korea; The School of Electrical and Information Engineering, The University of Sydney, NSW2006 Sydney, Australia

**Keywords:** Electrical impedance tomography (EIT), Field programmable gate array (FPGA), Application specific integrated circuit (ASIC), Biosignal-gated imaging, Fast multi-frequency imaging

## Abstract

**Background:**

Modern EIT systems require simultaneously operating multiple functions for flexibility, interoperability, and clinical applicability. To implement versatile functions, expandable design and implementation tools are needed. On the other hand, it is necessary to develop an ASIC-based EIT system to maximize its performance. Since the ASIC design is expensive and unchangeable, we can use FPGAs as a prior step to the digital ASIC design and carefully classify which functions should be included in the ASIC. In this paper, we describe the details of the FPGA design adopted in the KHU Mark2.5 EIT system.

**Methods:**

We classified all functions of the KHU Mark2.5 EIT system into two categories. One is the control and processing of current injection and voltage measurement. The other includes the collection and management of the multi-channel data with timing controls for internal and external interconnections. We describe the implementation of these functions in two kinds of FPGAs called the impedance measurement module (IMM) FPGA and the intra-network controller FPGA.

**Results:**

We present functional and timing simulations of the key functions in the FPGAs. From phantom and animal imaging experiments, we show that multiple functions of the system are successfully implemented in the FPGAs. As examples, we demonstrate fast multi-frequency imaging and ECG-gated imaging.

**Conclusion:**

Given an analog design of a parallel EIT system, it is important to optimize its digital design to minimize systematic artifacts and maximize performance. This paper described technical details of the FPGA-based fully parallel EIT system called the KHU Mark2.5 with numerous functions needed for clinical applications. Two kinds of FPGAs described in this paper can be used as a basis for future EIT digital ASIC designs for better application-specific human interface as well as hardware performance.

## Background

Electrical impedance tomography (EIT) is a technique for imaging conductivity distributions inside the human body by injecting currents and measuring induced voltages using electrodes on the surface [1–3]. Various EIT systems have been developed by several EIT research groups [3]. As the main component for waveform generation and phase-sensitive demodulation, the existing systems used either digital signal processor (DSP) [4–6] or field programmable gate array (FPGA) [7–13]. Though there have been attempts to design application specific integrated circuits (ASICs) for EIT [14], there is no ASIC-based EIT system yet available for imaging experiments.

Modern EIT systems require simultaneously operating multiple functions for flexibility and interoperability, which are needed in clinical applications. For example, in lung EIT for real-time ventilation and perfusion monitoring, we need ECG-gated imaging, fast multi-frequency imaging, interface to mechanical ventilator and patient monitor, and so on [15, 16]. To accomplish all of these, we need an EIT system with add-on functions beyond its basic imaging capability.

Development of a high-performance stable EIT system with numerous functions and high speed requires an iterative process to implement sophisticated digital as well as analog parts. For example, high-quality fast data collections in EIT require careful digital controls of timing signals, switches, and digital potentiometers, which are used together with analog circuits for current sources and voltage amplifiers [8, 17, 18]. These are especially important in a parallel EIT system to remove systematic artifacts and maximize performance since multiple current sources and voltmeters must be operating synchronously. Though there are various studies of analog techniques in EIT including current sources and voltage amplifiers [3, 19, 20], we found relatively little information on digital techniques in EIT. In this paper, we focus on the digital design of a fully parallel EIT system.

To implement versatile and expandable functions, it is desirable to use programmable and flexible tools during the system development stage. On the other hand, it is necessary to develop ASICs to maximize system performance for a given clinical application. Analog ASICs may improve the common-mode rejection ratio (CMRR), signal-to-noise ratio (SNR), reciprocity error (RE), and so on. We may implement numerous functions with reduced power consumption and physical size using digital ASICs. Such ASICs will also be useful for better human interface designs. ASIC designs, however, must be done carefully since the design process is expensive and unchangeable.

Lately, we have developed a fully parallel multi-frequency EIT system called the KHU Mark2.5 with various functions such as the automatic calibration, pipelining, and long-term stability [18]. Since its digital design was based on FPGAs, we could add new functions such as bio-signal gating and fast multi-frequency imaging using frequency multiplexing by changing only the FPGAs. Though Wi *et al* described the development of the KHU Mark2.5 EIT system [18], they focused on its overall structure and performance evaluation rather that providing technical details of the FPGAs.

In this paper, we describe the details of the FPGA design in the KHU Mark2.5 EIT system to implement all of its functions using two kinds of FPGAs. Carefully classifying and implementing the functions in the FPGAs, we considered future designs of EIT digital ASICs based on the FPGA designs. For ASIC-based EIT system developments, we will propose the FPGA design described in this paper as a basis of future EIT digital ASICs.

## Methods

### EIT system design using FPGAs

**Figure 1:**
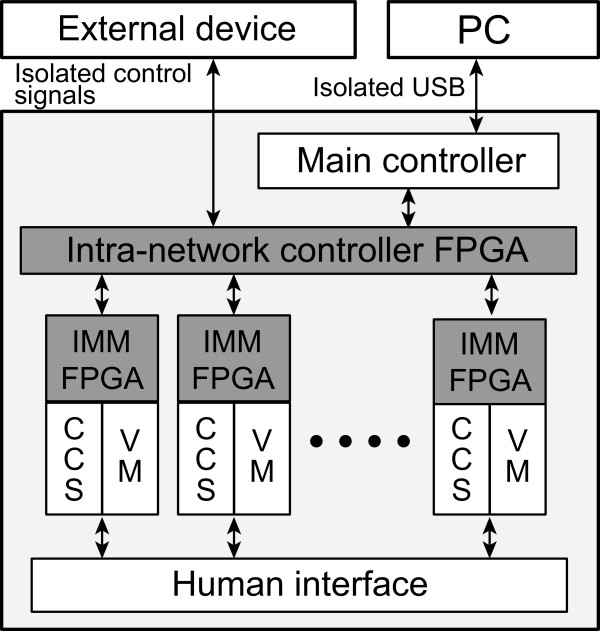
**Block diagram of the KHU Mark2.5 EIT system.** It consists of a DSP-based main controller, FPGA-based intra-network controller, and FPGA-based multiple IMMs. CCS and VM stand for constant current source and voltmeter, respectively.

Figure 1 shows the structure of the KHU Mark2.5 EIT system. It includes a DSP-based main controller, which is responsible for data communication with a PC through an isolated USB connection. There are multiple impedance measurement modules (IMMs) and each module includes a current source and a voltmeter. The intra-network controller arbitrates data exchanges among the main controller, IMMs, and external devices. We classified and implemented all functions of the system in two kinds of FPGAs; the intra-network controller FPGA and IMM FPGA.

### Intra-network controller FPGA

**Figure 2:**
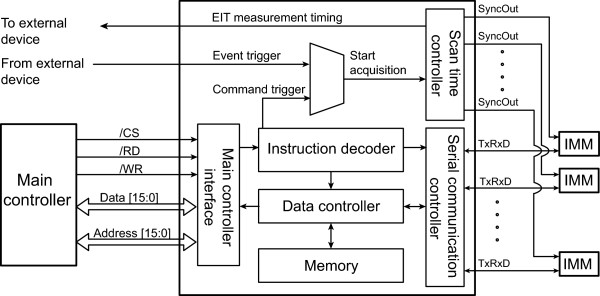
**Functional block diagram of the intra-network controller FPGA.** It handles all the data exchanges among the main controller, IMMs, and external devices. The intra-network controller connects to all IMMs through individual synchronous serial communication channels in the star network topology.

Figure 2 shows the functional block diagram of the intra-network controller FPGA. We can configure the EIT system with different numbers of IMMs and one intra-network controller FPGA can accommodate up to 32 IMMs. By adding one intra-network controller FPGA, the maximum number of IMMs is increased by 32.

The FPGA handles the command and data exchanges between the main controller and the connected IMMs. In designing the FPGA, we considered the data throughput to the main controller and the connected IMMs. Since the connection to the main controller requires fast exchanges of a large amount of data from all IMMs, we interfaced the FPGA to the main controller DSP in its external memory space.

In the KHU Mark2.5 EIT system, each IMM is independent and all of them operate in parallel. We, therefore, adopted the star network topology with a dedicated FIFO-based synchronous serial communication channel to each IMM, which is denoted as *TxRxD*. To implement the pipelined operation for a maximum frame rate of 100 frames/sec [18], we used the double-buffered memory structure in each serial communication channel to transmit previous data and store new data simultaneously.

Decoding the commands from the main controller, the intra-network controller FPGA may transmit its own commands to the IMMs through the serial communication channels and setup them for a chosen data collection protocol. When it gets a trigger command from the main controller, it initiates a series of data acquisitions by distributing timing synchronization signals called *SyncOut* to all connected IMMs. Though all IMMs are independent and operate in parallel, their data acquisitions are synchronized by sharing the common clock and timing signals from the FPGA. After triggering the IMMs for their data acquisitions, the intra-network controller FPGA receives the measured data from the IMMs through the serial communications channels.

In certain applications, it is highly desirable to synchronize EIT data acquisitions and image reconstructions with physiological events such as respiration and cardiac function. For example, ECG-gated EIT imaging may separate relatively small conductivity changes related with cardiac function from larger changes associated with lung ventilation [21]. In the external event trigger mode, the intra-network controller FPGA gets a sequence of external trigger signals to control the subsequent EIT data acquisitions. Each event trigger signal may initiate one or multiple data acquisitions by distributing the synchronization signals *SyncOut* to the connected IMMs.

Regardless of the operating mode, the intra-network controller FPGA can send the EIT measurement timing signal to external devices. The signal provides accurate timing information of EIT data acquisitions, which can be used to synchronize the external device with the EIT system. It may also be used to analyze measured data of the external device together with EIT images in a synchronized way. We implemented all the connections to external devices using the digital signal isolator (ADuM3100, Analog Devices, USA) with the low-voltage differential signaling (LVDS) technique for electrical safety and noise suppression.

### Impedance measurement module FPGA

**Figure 3:**
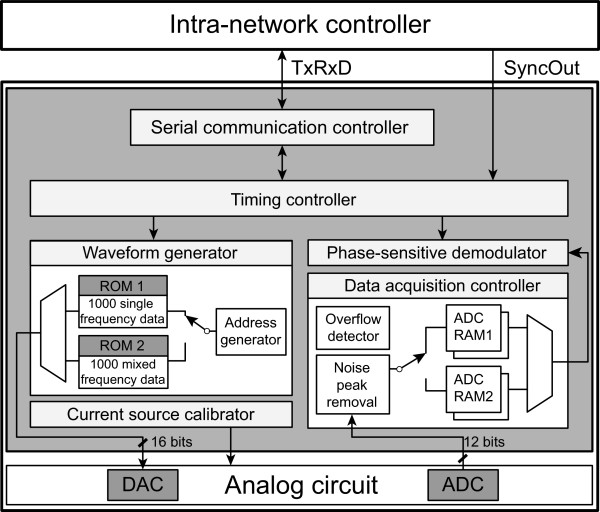
**Functional block diagram of the IMM FPGA.** Each IMM is independent of all others and all of them operate in parallel. The IMM FPGA controls and manages all functions of current injections and voltage measurements during real-time projections and scans. It also controls the timing of pipelined operations, switchings, and transient removals.

Figure 3 shows the block diagram of the IMM FPGA. It receives the synchronization signal *SyncOut* from the intra-network controller and exchanges commands and data via the dedicated serial communication channel *TxRxD*. Each IMM FPGA operates independently for current injection and voltage measurement regardless of the behavior of other IMMs [18].

We define a projection as one current injection and simultaneous multiple voltage measurements. At least two IMMs are involved in the current injection since we need at least one current source and at least one current sink to form a balanced current source. Usually, all IMMs are used to measure the induced voltages. A scan is a collection of projections to produce a set of measured data for one image reconstruction.

Real-time EIT data acquisitions include a series of scans with multiple projections in each scan. Therefore, we constantly change parameters of current injections such as electrodes, frequencies, and amplitudes. Accordingly, there occur transients in the induced voltage signals. To properly handle all of these switchings and transients in the digitized voltage signals as well as analog signals, the IMM FPGA must carefully implements all critical timing and functional controls of the real-time EIT data acquisition process.

#### Timing controller

Before starting any data acquisition, the intra-network controller provides a chosen data collection protocol as a protocol table to each IMM. The timing controller decodes the table and stores the commands and data in its memory space for the required projections and scans. These include the parameters for waveform generation, current source calibration, data acquisition, preprocessing, and switch control.

After receiving the synchronization signal *SyncOut* from the intra-network controller, the timing controller initiates a series of current injections and synchronous voltage data acquisitions. It generates various timing signals to coordinate all the operations of the IMM. Carefully adjusted timing controls are required to properly implement the pipeline operations [18] and the intermittent transient removals. For each projection, it gets the demodulated voltage data and transmits them to the intra-network controller through the serial communication channel *TxRxD*.

#### Waveform generator

We chose the memory-based waveform generation method using the register-transfer level (RTL) FPGA design. We used two internal ROMs to separately store 1,000 16-bit data of the single-frequency and mixed-frequency sinusoidal waveforms. The frequency is controlled by using two variables of *gapdata* and *clkcount* as
12

where *gapdata* determines the incremental step size of the ROM address, *t*_*update*_ is the address update time, and *S*_*clk*_ is the period of the system clock. We can choose frequencies in the range of 10 Hz to 500 kHz.

For each projection, the timing controller sends the waveform parameters including the chosen frequencies and amplitudes. The waveform generator reads the waveform data from the memory using the timing signals properly adjusted for the chosen sinusoidal frequency and sends the data to the external 16-bit DAC (AD9783, Analog Devices, USA). It also sets the registers of the DAC for its control.

The waveform generator sends a pulse called *Phase0* to the voltage demodulator. The pulse indicates the beginning of each sinusoidal period for the phase-sensitive demodulation of the induced voltage signal.

#### Current source calibrator

The output voltage of the DAC is converted to current using a trans-conductance amplifier, whose output impedance was calibrated beforehand to at least 1 M *Ω* in the entire frequency range [18]. To achieve the calibrated output impedance, we should properly set the digital potentiometers (DS1267E-010, -050, Maxim Integrated Products, Inc., USA) in the trans-conductance amplifier and also in the generalized impedance converter (GIC) at the output of the amplifier. Getting the values of the digital potentiometers extracted from the calibration table for the chosen frequencies, the current source calibrator sets up the digital potentiometers before current injection in each projection. It also sets the multiplying DAC (AD9783, Analog Devices, USA) to adjust the amplitude of the sinusoidal voltage waveform to reduce the dc offset current within ±33 nA.

During the system calibration beforehand, the current source calibrator iteratively adjusts the digital potentiometers to find their values to achieve at least 1 M *Ω* output impedance. As described by Wi *et al* [18], this should be done with the voltmeter calibration as well. During real-time operations, the current source calibrator keeps the calibration table in its memory space. The voltmeter calibrations to compensate phase and gain errors are not included in the IMM but in the system software of the PC.

#### Data acquisition controller

The data acquisition controller provides the control signals to the external 12-bit ADC (AD9235, Analog Devices, USA). To continuously read ADC data, it uses the double buffered structure with two internal RAMs for real-time pipelined data acquisitions. When one of the RAMs is full with a predetermined amount of data, it is connected to the phase-sensitive demodulator and the other RAM is used to store the next set of data. The 12-bit ADC data is sign-extended to the 18-bit representation and the data acquisition controller performs chosen real-time preprocessing functions such as overflow detection, noise peak removal, and averaging of maximum 64 sinusoidal periods of data.

#### Phase-sensitive demodulator

The phase-sensitive demodulator computes the in-phase (real-part) and quadrature (imaginary-part) components, *V*_*r*_ and *V*_*q*_, respectively, of the induced voltage *V*_*in*_ as
34

During the phase-sensitive demodulation, the 18-bit sign-extended ADC data are multiplied with the 16-bit sinusoidal data stored in the ROM of the waveform generator. To perform 1,000 34-bit additions, we used the 44-bit arithmetic architecture to prevent overflow. It is important to carefully allocate enough memory space to prevent data loss during fast real-time demodulations.

When we measure the induced voltage at multiple frequencies, the phase-sensitive demodulations are simultaneously performed for maximum three different frequencies. For more than three frequencies, the demodulations are repeated using the same acquired ADC data and the stored sinusoidal waveform data at those frequencies.

### Functional and timing simulations of FPGAs

We used the software package Quartus II (Altera, USA) for the FPGA design and simulation. For the chosen FPGA (EP3C10F256, Altera, USA) at 45 MHz clock speed, we verified the design for its functions and timing. We assumed that the operating frequency of the EIT data acquisition was 11.25 kHz. We also assumed that current was injected between one neighboring electrode pair and induced voltages were measured between all neighboring electrode pairs. After finishing the simulation of each part separately, we simulated the complete FPGA design including both the intra-network controller FPGA and IMM FPGAs.

### Phantom experiments of fast multi-frequency imaging

**Figure 4:**
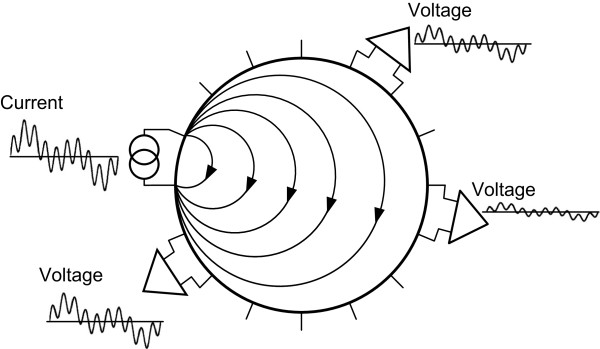
**Conceptual diagram of multi-frequency waveform multiplexing.** As an example, the mixed sinusoidal waveform with three different frequencies is used for current injection. Phase-sensitive demodulations of the induced voltage signals at the chosen frequencies are performed in parallel.

Multi-frequency imaging requires fast data acquisitions at chosen operating frequencies. In the multi-frequency waveform multiplexing method [4], currents are injected in a waveform of mixed sinusoids with multiple frequencies. Figure 4 shows an example of injecting current with three different frequencies. Each voltmeter simultaneously demodulates the induced voltages at those three frequencies at the same time. We used three frequencies considering the safe levels of injection currents and SNRs of induced voltages at chosen frequencies [22].

### Animal experiments of ECG-gated imaging

**Figure 5:**
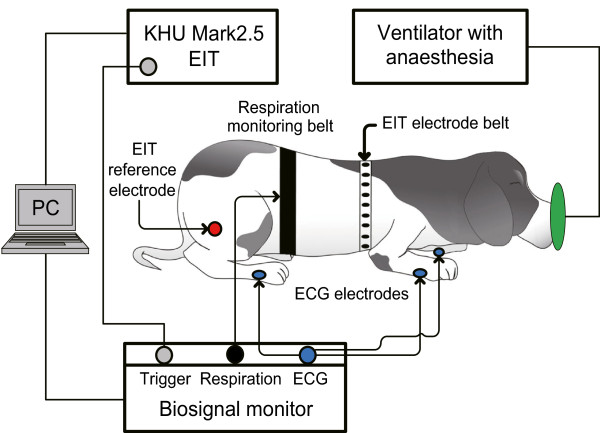
**Animal experimental set-up.** The experimental setup for monitoring of cardiac cycles and respiration with the ECG-gated EIT images on the beagle.

We performed *in vivo* animal experiments using a beagle (9 years old, female, 13 kg). The experimental protocol was approved by the Institutional Animal Care and Use Committee at Kyung Hee University. Putting the animal under anesthesia following the process described in Oh *et al* [23], we shaved the hair around the chest and attached the EIT electrode belt with 16 Ag/AgCl electrodes. We also attached a separate respiration monitoring belt and ECG electrodes as shown in Figure 5. The experimental protocol was approved by the Institutional Animal Care and Use Committee at Kyung Hee University (KHUASP(SU)-11-07).

We used a custom designed device to acquire ECG and respiration signals at 500 Hz sampling frequency. Detecting R waves of the acquired ECG signal, we triggered a series of EIT scans at each R wave. We also triggered a series of EIT scans at a predetermined phase of each respiration cycle.

## Results

### FPGA simulation results

**Figure 6:**
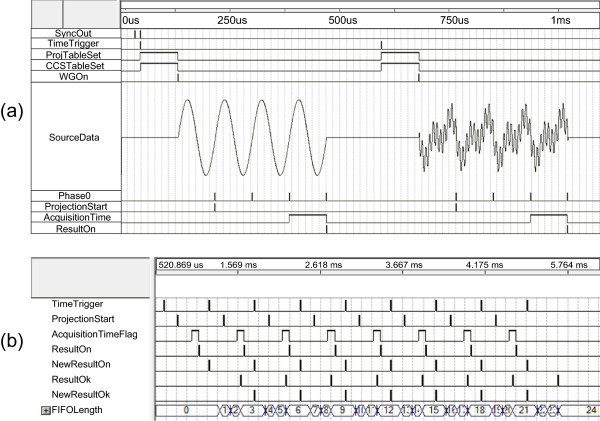
**Functional and timing simulations of the intra-network controller FPGA and IMM FPGA.** **(a)** Simulation of two consecutive projections using single-frequency and multi-frequency injection currents. **(b)** Timing simulation of pipelined data acquisitions and phase-sensitive demodulations.

Figure 6(a) and (b) illustrate FPGA simulation results for 11.25 kHz sinusoidal waveform generation and data acquisition, respectively. The time for one projection was 0.58 ms. In Figure 6(a), the intra-network controller FPGA generated two consecutive *SyncOut* pulses. The first pulse was used to reset the counters and registers in the IMM FPGA. The second pulse produced *TimeTrigger* signal, which set the reference time for each projection. It also initiated parameter table settings for the projection. The *WGOn* signal indicated that the waveform generator was ready to start the requested waveform generation. The signal denoted as *SourceData* was the generated sinusoidal waveform at 11.25 kHz.

Starting at the beginning of the second period, the *Phase0* signals were produced at every cycle of the waveform. The *ProjectionStart* signal was generated at the first *Phase0* signal and properly set all analog switches. The actual data acquisition was delayed by two *Phase0* signals to provide enough time for the injection current and induced voltage to be stabilized. Finishing the first projection with single-frequency current injection, the second projection was started using the mixed frequency waveform.

Figure 6(b) shows the timing details of the data acquisition from the ADC. Within each projection, the ADC acquired the voltage data during the *AcquisitionTimeFlag* signal. Finishing the data acquisition, the *ResultOn* signal was generated, which turned the phase-sensitive demodulator on. The *ResultOk* signal indicated the end of the demodulation. Two other signals of *NewResultOn* and *NewResultOk* were used to implement the pipelined operations. The *FIFOLength* signal indicated the size of the demodulation data stored in the FIFO. The timing controller in the IMM FPGA read the data and sent them to the intra-network controller FPGA.

### Fast multi-frequency images of phantom

**Figure 7:**
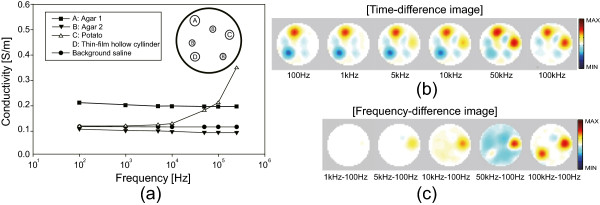
**Multi-frequency imaging of phantom.** **(a)** Configuration of six objects in the saline phantom and their conductivity spectra. **(b)** Time-difference images simultaneously acquired at six different frequencies of 0.1, 1, 5, 10, 50, and 100 kHz. **(c)** Frequency-difference images between five different frequencies of 1, 5, 10, 50, 100 kHz and 100 Hz are displayed. Here, we used the image at 100 Hz as the reference for all pairs.

Figure 7(a) shows the configuration of the saline phantom and measured conductivity spectra of the objects using the impedance analyzer (SI1260, Solatron, UK). We chose two sets of three frequencies including (0.1, 1, 5) kHz and (10, 50, 100) kHz. We could reduce the data acquisition time by 9.2% for the first set of frequencies. Amplitudes of the currents at those frequencies were adjusted to be within the safety limits [22]. We used the GREIT algorithm [24] to produce time- and frequency-difference images of the phantom.

To better understand the reconstructed multi-frequency images, we summarize the conductivity distribution of the phantom, of which background was 0.12 Sm ^-1^ saline. The object A and B were agar gels with 0.22 and 0.11 Sm ^-1^, respectively, over the entire frequency range. The object C was a cylindrical piece of potato with 30 mm diameter. Its conductivity increased from 0.12 to 0.35 Sm ^-1^ at 100 Hz and 500 kHz, respectively. The object D was a thin-film hollow cylinder. Though we did not measure the conductivity spectrum of the object D, we can predict that its effective conductivity was very small at low frequencies and increased to a measurable value at high frequencies.

In Figure 7(b), the objects A and B appeared in all time-difference conductivity images from 100 Hz to 100 kHz since their conductivity values were constant over the frequency range. The object C (D) appeared in the time-difference images at high (low) frequencies and disappeared at low (high) frequencies since their conductivity values changed with frequency. Figure 7(c) shows the reconstructed frequency-difference images, which can be interpreted similarly.

### Ventilation and ECG-gated images of animal

**Figure 8:**
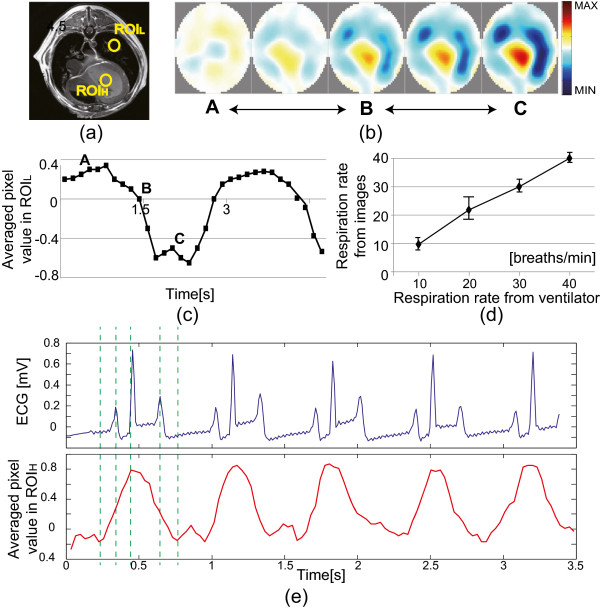
**Animal imaging with biosignals.** **(a)** Axial MR image of the beagle at the plane of the attached EIT electrodes. **(b)** Time series of time-difference images at 11.25 kHz during mechanical ventilation. **(c)** Plot of the average pixel values of the ROI _*L*_ inside the lung region. **(d)** Estimated respiration rate from the reconstructed images is well correlated with the ventilator setting. **(e)** Plot of the average pixel values of the ROI _*H*_ inside the heart region, which is well correlated with the ECG signal. ECG-gating imaging was conducted to produce these results.

As shown in Figure 8(a), we defined two regions of interest (ROIs) marked as ROI_*L*_ and ROI_*H*_ in the lung and heart regions, respectively. We used the mechanical ventilator (M-2002, Hallowell EMC, Pittsfield, MA, USA) to vary the respiration rate from 10 to 40 breaths/min. Figure 8(b) shows the typical EIT images of the chest at 20 breathes/min respiration rate. Figure 8(c) plots a time series of the average pixel values in ROI_*L*_, which distinguished different phases of respiration cycles. Figure 8(d) shows that the estimated respiration rates from the reconstructed images matched well with those of the mechanical ventilator.

Figure 8(e) shows the ECG signal used for ECG-gated imaging and the time series of the average pixel values in ROI_H_. The changes of the pixel values well correlated with the ECG signal. From the pixel values in the heart region, we could observe about 3.2 dB (16.8%) SNR improvement in the ECG-gated images compared with those of conventional time-difference images. When we evaluated the SNR of the pixel values in the lung region, we could observe about 6 dB (13.9%) improvement when we acquired the ventilation images at a fixed time within every cardiac cycle.

## Discussions

We have adopted the FPGA-based design method from the development of the KHU Mark1 EIT system to the Mark2.5 system [8, 17, 18]. We could enhance the performance and add new functions by improving the FPGA designs with a minimal amount hardware changes. For example, the maximum frame rate increased from 6 in the Mark1 system to 100 frames/sec in the Mark2.5 system. The SNR, RE, and stability were also improved by carefully adjusting the complicated timing signals to remove the adverse effects of switchings and transients. The KHU Mark2.5 system now provides versatile functions such as reconfiguration of channels, automatic calibration, arbitrary current injection patterns, data averaging, fast multi-frequency imaging, external triggering, timing signal output, and isolated interface with external devices. All of these functions add flexibility and interoperability which are needed for clinical applications. We believe that the FPGA designs in the KHU Mark2.5 system are fully tested and can be a basis for future EIT digital ASIC designs.

We would like to highlight the parallel processing architecture of multiple independent IMMs. The intra-network controller can separately send each IMM its command tables including all the parameters of current injections and voltage measurements during a series projections and scans. During the development cycle, this actually added a significant amount of complexity in the FPGA designs especially for the implementation of the pipeline operation throughout the parallel system. The parallel processing architecture, however, now allows us to easily implement any data collection protocols needed in different applications.

The KHU Mark2.5 system with its parallel processing architecture is, however, bulky and heavy even though it has clear advantages in terms of speed and flexibility. It also requires a large amount of power consumption due to a large number of components. For most image-based clinical monitoring applications, it is highly desirable to reduce its total power consumption as well as physical size and weight. In addition, the human interface must be greatly simplified by placing more electronics on or near electrodes.

To address these practical issues, we plan to develop two digital ASICs. The first will be a main controller ASIC, which will combine the DSP and the intra-network controller described in this paper. Using this digital ASIC, we will be able to connect the future EIT system to a PC or mobile device through wired or wireless communication channels. The second digital ASIC will include all other digital parts, ADC, and DAC to implement the functions of the IMM FPGA. We speculate that future EIT digital ASIC designs will take advantage of the current fully tested FPGA designs.

In this paper, we focused on the FPGA designs which handle all the functions implemented by the digital technology. Though we tried to minimize the analog circuits in the KHU Mark2.5 system, its performance is now mostly influenced by the low-noise analog circuits and the human interface part. To further improve the system performance, it will be also desirable to develop an analog ASIC including a trans-conductance amplifier for current output and a differential voltage amplifier for voltage input.

## Conclusions

Modern EIT systems require simultaneously operating multiple functions for flexibility, interoperability, and clinical applicability. In this paper, we described technical details of two kinds of FPGAs, which implemented all functions of the fully parallel multi-frequency EIT system called the KHU Mark2.5. We chose the FPGA-based method to take advantage of its programmability, which was needed during the development cycle. To further maximize the performance and minimize the size and weight of the system for a specific clinical application, we plan to develop an ASIC-based system. Since the ASIC design process is expensive and unchangeable, we suggest using the developed FPGAs as a test bed for future EIT digital ASIC designs.

## Abbreviations

ECG: Electrocardiography
FPGA: Field programmable gate array
DSP: Digital signal processor
EIT: Electrical impedance tomography
IMM: Impedance measurement module
USB: Universal serial bus
RAM: Random access memory
CCS: Constant current source
VM: Voltmeter
FIFO: First in first out
PCB: Printed circuit board
ADC: Analog to digital converter
DAC: Digital to analog converter
SNR: Signal to noise ratio.
